# Prognostic significance and immune characteristics of GPR27 in gastric cancer

**DOI:** 10.18632/aging.205023

**Published:** 2023-09-12

**Authors:** Jun Pan, Yuanjun Gao

**Affiliations:** 1 Department of Gastroenterology, Taihe Hospital, Hubei University of Medicine, Shiyan 442000, Hubei, China

**Keywords:** gastric cancer, G-protein-coupled receptor 27, immunotherapy, survival

## Abstract

Gastric cancer (GC) is one of the most typical cancerous neoplasms occurring in the digestive system. For advanced GC, immunotherapy is the final option for them to prolong survival time. Hence, we aimed to identify new molecular targets to enhance the immunotherapy response in GC individuals. Then we applied bioinformatic analysis to explore the expression profiles of G-protein-coupled receptor 27 (GPR27) transcription and GPR27 methylation. The associations between survival of GC patients and GPR27 transcription and methylation were then analyzed. We also studied the link between GPR27 expression and levels of immune cell infiltration. Finally, we gained insights into the prognostic role of GPR27 protein in 97 cases of GC individuals. According to datasets gained from TCGA, GPR27 mRNA is expressed lower in GC tissues. Down-regulation of GPR27 transcription was related with better survival in GC individuals, and GPR27 cg03024619 had the most significant prognostic value (HR=0.553, P<0.0001). In addition, the expression level of GPR27 has a clear interaction with immune cells' infiltration and their markers. Single-cell analysis displayed that GPR27 is mainly expressed in macrophages. Finally, down-regulation of GPR27 protein was observed in GC tissues and correlated with better survival outcomes. GPR27 can serve as an important prognostic biomarker and exert an immunomodulatory role in GC. Our findings highlight the significance of GPR27 in a variety of cancers, including GC, and provide clues for a better understanding of GPR27 from bioinformatics and clinically validated perspective.

## INTRODUCTION

Gastric cancer (GC) is one of the most typical cancerous neoplasms occurring in the digestive system, and it represents the second most well-known reason for cancer death worldwide [1]. The common risk factors for GC include the presence of *Helicobacter pylori*, genetic susceptibility and diets high in nitrates and nitrites [2]. Surgical resection, systemic chemotherapy, targeted therapy, and radiotherapy are all proven to be efficient in GC treatments [3], but the long-term survival outcomes of GC sufferers are far from satisfactory, especially in patients with advanced GC. For advanced GC patients, immunotherapy is the final option for them to prolong survival time [4, 5], but fewer individuals could benefit from this novel therapy. Hence, identification of new molecular targets to enhance the immunotherapy response in GC individuals is an urgent need.

G-protein-coupled receptors (GPCRs) are a super-family with more than 800 members, and some of them are not well studied [6]. G-protein-coupled receptor 27 (GPR27) is a newly identified component of GPCRs super-family. A previous study reported that GPR27 is implicated in key physiological functions, such as energy metabolism, and insulin secretion and regulation, and neuronal plasticity [7]. Recent studies have linked GPR27 to the development and progression of malignancy. Wang et al. [8] suggested that GPR27 contributes to the proliferation of cancerous liver cells via the regulation of the MAPK/ERK signaling pathway and S phase entry. Moreover, Malin et al. and his coworkers [9] reveal that GPR27 is a methylation-driven gene, which may contribute to the occurrence and metastasis of cervical cancer. However, there is no information about GPR27 in GC.

In our research, we firstly examined the expression profile of GPR27 in various human tumors and normal tissues, then measured the potential correlation between GPR27 mRNA expression and its methylation levels, and investigated its correlation with mutations and tumor mutational burden (TMB). Besides, we delved into the correlation between GPR27 and GC patient survival according to sequencing data gained from TCGA dataset, and further confirmed this survival correlation with sequencing data from the GEO database. Subsequently, we analyzed the relationship between GPR27 expression and the immune microenvironment and potential biological pathways in GC. Finally, we used clinical cohort data (N=97) to verify the relationship between GPR27 protein levels and survival in GC patients.

## MATERIALS AND METHODS

### Xena Shiny

Xena Shiny is an easy-to-use database for quickly searching, analyzing and visualizing data from UCSC Xena data hubs [10]. We used Xena Shiny to explore the expression of GPR27 in pan-cancer and its association with TMB and MSI.

### GEPIA 2

GEPIA 2 is a web-based tool for in-depth analysis of transcriptome data in TCGA database [11]. It was used to investigate GPR27 expression in GC.

### MethSurv

MethSurv [12], is a network tool for multivariate survival analysis using DNA methylation data, which we used to explore survival and correlation between 12CpG and GPR27 gene methylation. We defined patients as high groups and low groups according to methylation medians.

### cBioPortal

cBioPortal contains large-scale cancer genomics datasets and provides capabilities including visualization, downloading, and analysis [13]. cBioPortal was used to explore mutation of GPR27 in GC and survival analysis of GC patients between GPR27 mutation group and unaltered group.

### Kaplan–Meier plotter

KM plotter was applied to investigate the prognostic worth of GPR27 in GC [14]. GC patients were defined as high and low groups according to median GPR27 level, then the HRs, 95% CIs, and logarithmic rank P values of overall survival (OS) and disease-free survival (DFS) were examined.

### TIMER

TIMER was applied for investigation of various immune cells’ infiltration levels [15]. Firstly, we applied the “Diff Exp” module to examine the expression level of GPR27 in all human malignancies. Then we used the “Gene” module to examine the correlation between GPR27 mRNA level and infiltration levels of immune cells using the dataset we gained from TCGA. Finally, we applied the “Correlation” module to examine the correlation between GPR27 mRNA level and immune cells’ markers.

### TISIDB

TISIDB is a website for gene-immune and tumor-immune interaction analysis [16]. It was applied to investigate GPR27 mRNA level in different molecular subtypes and immune subtypes.

### ImmuneCellAI

The Immune Cells Abundance Identifier (ImmuCellAI) is a novel network tool for assessing the number of immune cells, focusing on T cell subsets associated with tumor progression and elicitation [17]. Using transcription data from GC in TCGA, the distinction in abundance of 24 immune cells in the low group and high group of GPR27 was estimated.

### The human protein atlas

HPA is a public database launched in 2003 that uses a variety of holographic techniques to detect various human proteins. This database consists of 10 separate sections, and we explored the Single Cell section to identify which immune cells express GPR27 via single-cell sequencing analysis.

### Collection of GC tissues

We purchased a total of 180 paraffin-embedded tissue arrays (HStmA180Su19-M-066) from Shanghai Outdo Biotech Co, Ltd, including 97 gastric tumors and 83 normal tissues, with 5-year follow-up information. All clinical samples were clustered under patients’ informed consent. Our study plan was checked and accepted by the Ethics Committee of Taihe Hospital of Hubei University of Medicine (2022KS44) and performed based on the Declaration of Helsinki.

### Immunohistochemical

Immunohistochemistry (IHC) staining was conducted to explore GPR27 protein levels in gastric cancerous tissues and adjacent gastric normal tissue as reported [18]. Firstly, we deparaffinized the tissue sections by immersing them in xylene and rehydrating them using a series of graded alcohols. Then, we performed antigen retrieval to enhance antigen accessibility. We heated the sections in a 10mM citrate buffer (pH 6.0) for 20 mins. Subsequently, we blocked the sections with 3% H_2_O_2_ solution to prevent nonspecific binding. We incubated the tissue sections with anti-GPR27 protein antibody (1:300 dilution, No. bs-13528R, Bioss, Beijing, China) in 4° C refrigerators overnight. Then we washed the sections multiple times with PBS buffer to remove any unbound antibodies. Next, we incubated the tissue section with the secondary antibody. We developed the sections using 3, 3’- diaminobenzidine (DAB) and counterstained the sections with hematoxylin.

### Immunohistochemical scoring

IHC scoring is a method used to quantify the staining intensity and distribution of a target protein in tissue samples. We applied a semi-quantitative scale in our study that takes into account staining intensity and staining scope. Positive staining of GPR27 protein refers to cytoplasmic staining of GC cells. Staining intensity may be categorized as 0 point (absent), 1 point (weak), 2 points (moderate), and 3 points (strong), while the staining scope can be categorized as 1 point (1%–25%), 2 points (26%–50%), 3 points (51%–75%), and 4 points (76%–100%). The final IHC scores of each tissue were obtained by the multiplication of the scores for staining intensity and staining scope. Then we defined GC patients as low GPR27 protein and high GPR27 protein groups according to the median value of GPR27 protein level in all those GC patients.

### Statistical analysis

Our analysis was conducted using SPSS software (version 21) and R software (version 3.5.1). Student’s t-tests were applied to examine the statistical significance of the GPR27 immunohistochemical (IHC) scores between GC tissues and adjacent tissues. To assess the correlations between GPR27 mRNA expression and clinical characteristics of GC, we utilized either Chi-square or Fisher’s exact test, depending on the specific circumstances. To present the associations between survival outcomes and GPR27 mRNA, DNA methylation, and protein expression, we utilized Kaplan-Meier survival analysis. A P value of less than 0.05 was considered statistically significant.

### Data availability statement

The datasets used and analyzed during the current study are available from the corresponding author on reasonable request.

## RESULTS

### GPR27 has low expression in several cancerous tissues

**Figure 1:**
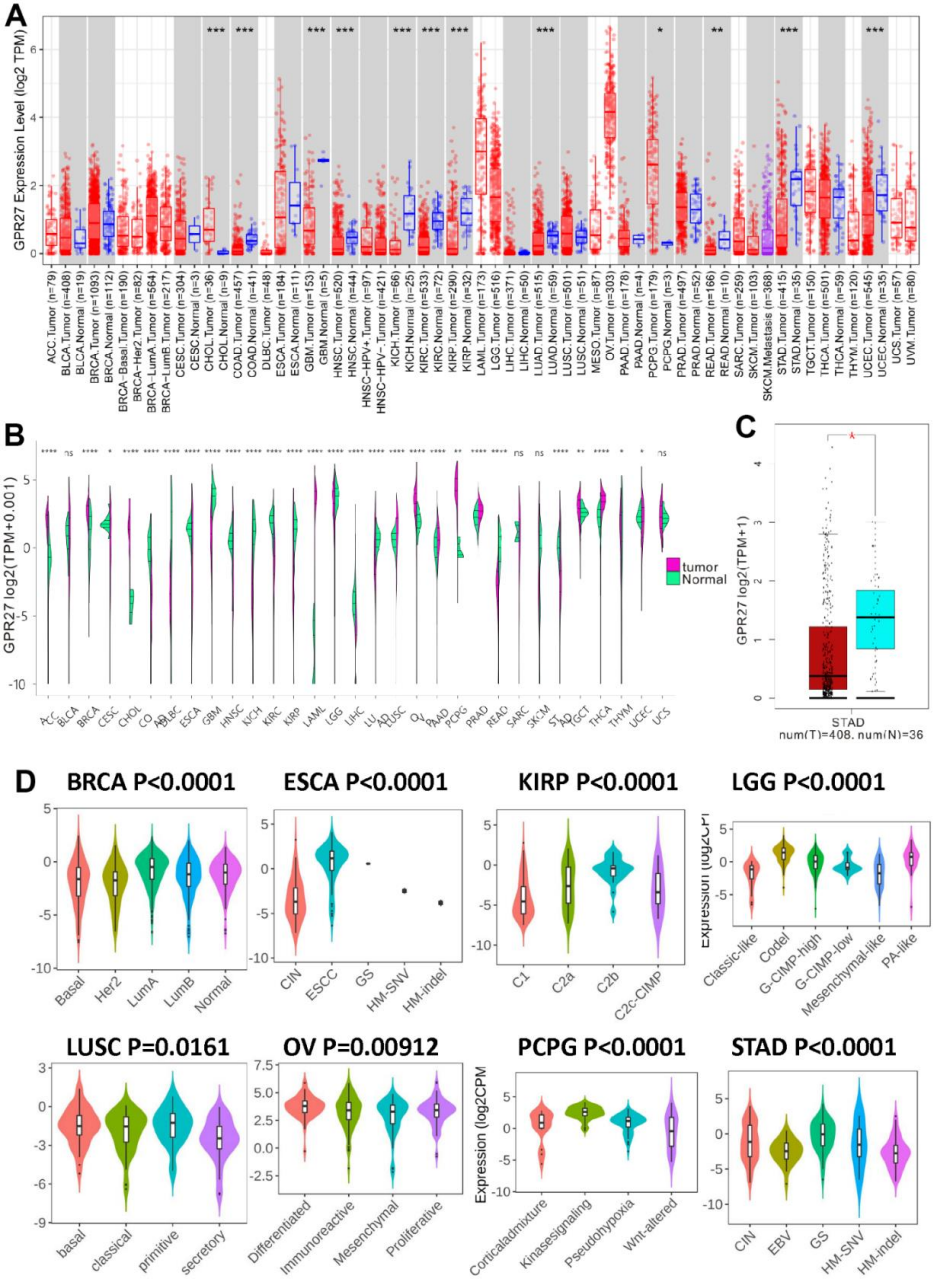
**The transcription levels of GPR27 in human cancers.** GPR27 mRNA expression in pan-cancer from TIMER (https://cistrome.shinyapps.io/timer/) (**A**) and Xena Shiny (https://shiny.hiplot.com.cn/ucsc-xena-shiny/) (**B**) and gastric cancer (**C**). The expression of GPR27 in different molecular subtypes of cancers via TISIDB (http://cis.hku.hk/TISIDB/index.php) (**D**).

Firstly, we investigated GPR27 mRNA expression level in various human cancers through TIMER (Figure 1A) and Xena Shiny (Figure 1B). Results show that GPR27 is lowly expressed in several cancerous tissues including STAD, COAD, READ, LUAD, GBM, KIRC, KICH, KIRP, HNSC and UCEC. By contrast, GPR27 mRNA was highly expressed in CHOL and PCPG. Then we investigated the GPR27 mRNA levels between GC tissues and normal gastric mucosa basing on data gained from TCGA dataset, and results show that GPR27 mRNA was down-regulated in GC tissues than in normal gastric mucosa (Figure 1C). GPR27 mRNA level in different molecular subtypes of STAD, ESCA, KIRP, LGG, LUSC, BRCA, OV and PCPG were significantly different (Figure 1D).

### GPR27 mutation correlates with GPR27 expression and GC patients’ survival

cBioPortal was applied to analyze genetic alteration of GPR27 in GC patients. The results show that missense mutation of GPR27 was found in 4% of GC patients (Supplementary Figure 1A–1C). The mutation of GPR27 was well correlated with some clinical indices, such as TMB and gender (Supplementary Table 1). Next, we explored the correlation between GPR27 mutation and GC patients’ survival. We found that GC individuals with GPR27 alteration showed worse OS (log rank P=0.0103, Supplementary Figure 1D) and DFS (log rank P=0.0116, Supplementary Figure 1E) than those without GPR27 alteration.

### GPR27 mRNA expression level correlates with GC patients’ survival

**Figure 2:**
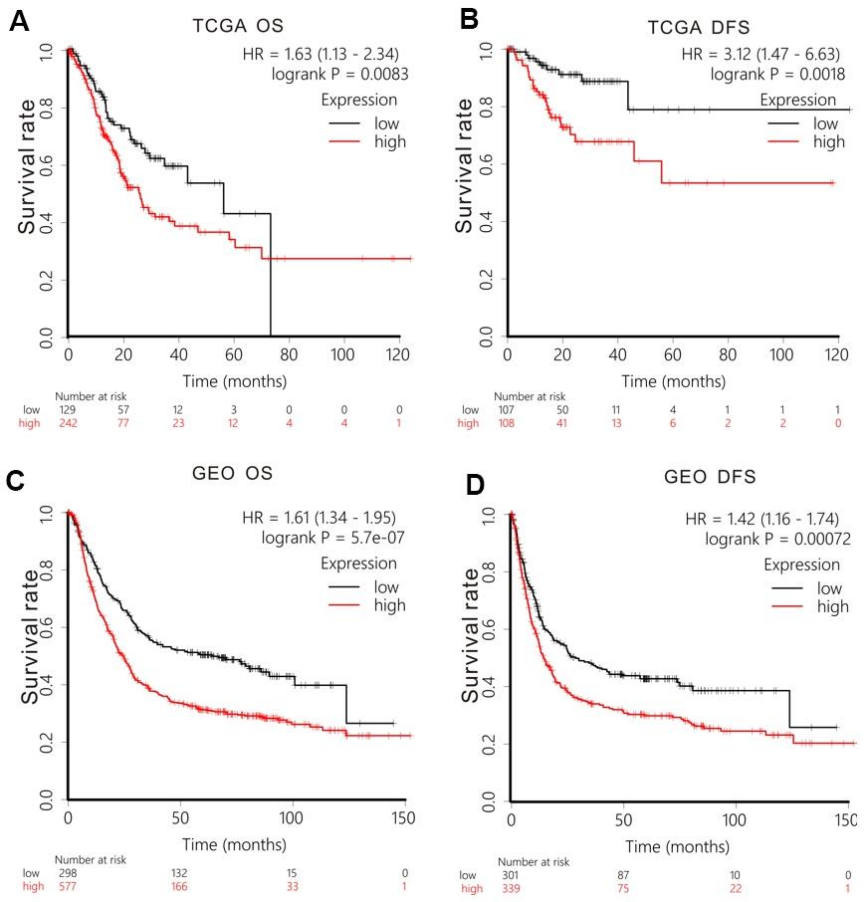
**Survival analysis of GPR27 mRNA in gastric cancer.** Low levels of GPR27 are correlated with longer overall time (**A**) and disease-free survival time (**B**) based on TCGA dataset. Low levels of GPR27 are related to longer overall time (**C**) and disease-free survival time (**D**) based on KM Plotter (http://kmplot.com).

We examined the prognostic worth of GPR27 in GC via K-M Plotter. Firstly, we analyzed TCGA-STAD cohort and found that GC patients with lower level of GPR27 had a longer OS (HR=1.63, 95%CI:1.13-2.34, P=0.0083, Figure 2A) and DFS (HR=3.12, 95%CI:1.47-6.63, P=0.0018, Figure 2B). To further authenticate our conclusion, we also conducted survival analysis in GEO cohort. Based on 875 GC patients, we gained a similar conclusion: GC patients with lower expression of GPR27 exhibited longer OS (HR=1.61, 95%CI:1.34-1.95, P=5.7e-07, Figure 2C) and DFS (HR=1.42, 95%CI:1.16-1.74, P=0.00072, Figure 2D). Furthermore, we examined the association between GPR27 mRNA level and survival in GC patients with various clinical metrics using Kaplan-Meier plotter (Table 1).

**Table 1:** Correlation of GPR27 expression and survival in gastric cancer with different clinical metrics by Kaplan-Meier plotter.

**Clinical features**	**N**	**HR**	**Overall survival**		**Disease-free survival**		
			**95%CI**	**P value**	**HR**	**95%CI**	**P value**
**Gender**							
female	244	1.8	1.27-2.56	0.00084	1.59	1.08-2.34	0.018
male	566	1.83	1.43-2.35	1.4*e-6	1.56	1.22-1.99	0.00031
**Proliferation**							
No	169	0.7	0.47-1.04	0.077	0.71	0.48-1.04	0.79
Yes	4	-	-	-	-	-	-
**Treatment**							
Surgery	393	1.25	0.93-1.68	0.14	0.86	0.63-1.18	0.36
Chemotherapy	157	0.73	0.49-1.08	0.12	0.71	0.48-1.05	0.084
Other adjuvant	80	0.53	0.21-1.29	0.15	0.43	0.19-0.97	0.037
**Her2 status**							
Negative	641	1.8	1.43-2.27	3.6*e-7	1.56	1.12-2.03	0.00079
Positive	424	1.19	0.9-1.57	0.23	1.23	0.86-1.76	0.26
**T stage**							
T1	14	-	-	-	-	-	-
T2	253	1.18	0.72-1.94	0.5	1.2	0.79-1.81	0.39
T3	208	1.49	1.04-2.14	0.031	0.72	0.49-1.06	0.093
T4	39	1.43	0.52-3.9	0.48	0.46	0.18-1.14	0.084
**N stage**							
N0	76	4.62	1.08-19.75	0.023	4.64	1.04-19.1	0.027
N1-3	437	1.35	1.04-1.76	0.025	0.74	0.56-0.98	0.036
**M stage**							
M0	459	1.35	1.01-1.79	0.039	1.18	0.91-1.54	0.22
M1	58	1.73	0.94-3.19	0.077	1.55	0.84-2.86	0.16
**TNM stage**							
Stage I	69	5.88	0.77-44.74	0.052	-	-	-
Stage II	145	1.4	0.77-2.55	0.27	1.39	0.75-2.59	0.29
Stage III	319	1.72	1.28-2.3	0.00026	1.33	0.89-1.99	0.16
Stage IV	152	1.36	0.93-2.0	0.1	0.72	0.48-1.07	0.1
**Lauren classification**							
Diffuse	248	1.34	0.95-1.9	0.095	1.25	0.86-1.81	0.24
Intestinal	336	1.85	1.34-2.54	0.00012	1.28	0.9-1.82	0.16
Mixed	33	3.07	0.86-10.91	0.068	0.49	0.14-1.73	0.26
**Differentiation**							
poor	166	0.72	0.48-1.08	0.11	0.61	0.38-0.97	0.037
moderate	67	1.34	0.64-2.79	0.43	0.63	0.31-1.28	0.2
well	32	2.58	0.76-8.82	0.12	-	-	-

### Subgroup survival analysis stratified by tumor mutation burden (TMB) in GC

Xena Shiny was used to calculate TMB for each sample. Then we analyzed correlations between GPR27 mRNA level and TMB, and GPR27 methylation level and TMB. The results show that the GPR27 mRNA level is inversely linked to TMB (r=-0.267, P<0.0001, Supplementary Figure 2A), while GPR27 methylation level is positively linked to TMB (r=0.327, P<0.0001, Supplementary Figure 2B). In survival analysis of low-TMB group, GC patients with lower expression of GPR27 displayed longer OS (HR=1.34, 95%CI:0.85-2,13, P=0.21, Supplementary Figure 2C) and DFS (HR=2.52, 95%CI:1.02-6.21, P=0.037, Supplementary Figure 2D). While high TMB group survival analysis showed the same results: GC patients with lower expression of GPR27 exhibited longer OS (HR=1.76, 95%CI:1.08-2.85, P=0.02, Supplementary Figure 2E) and DFS (HR=3.5, 95%CI:1.27-9.61, P=0.01, Supplementary Figure 2F).

### Epigenetic regulation of GPR27 in GC

**Figure 3:**
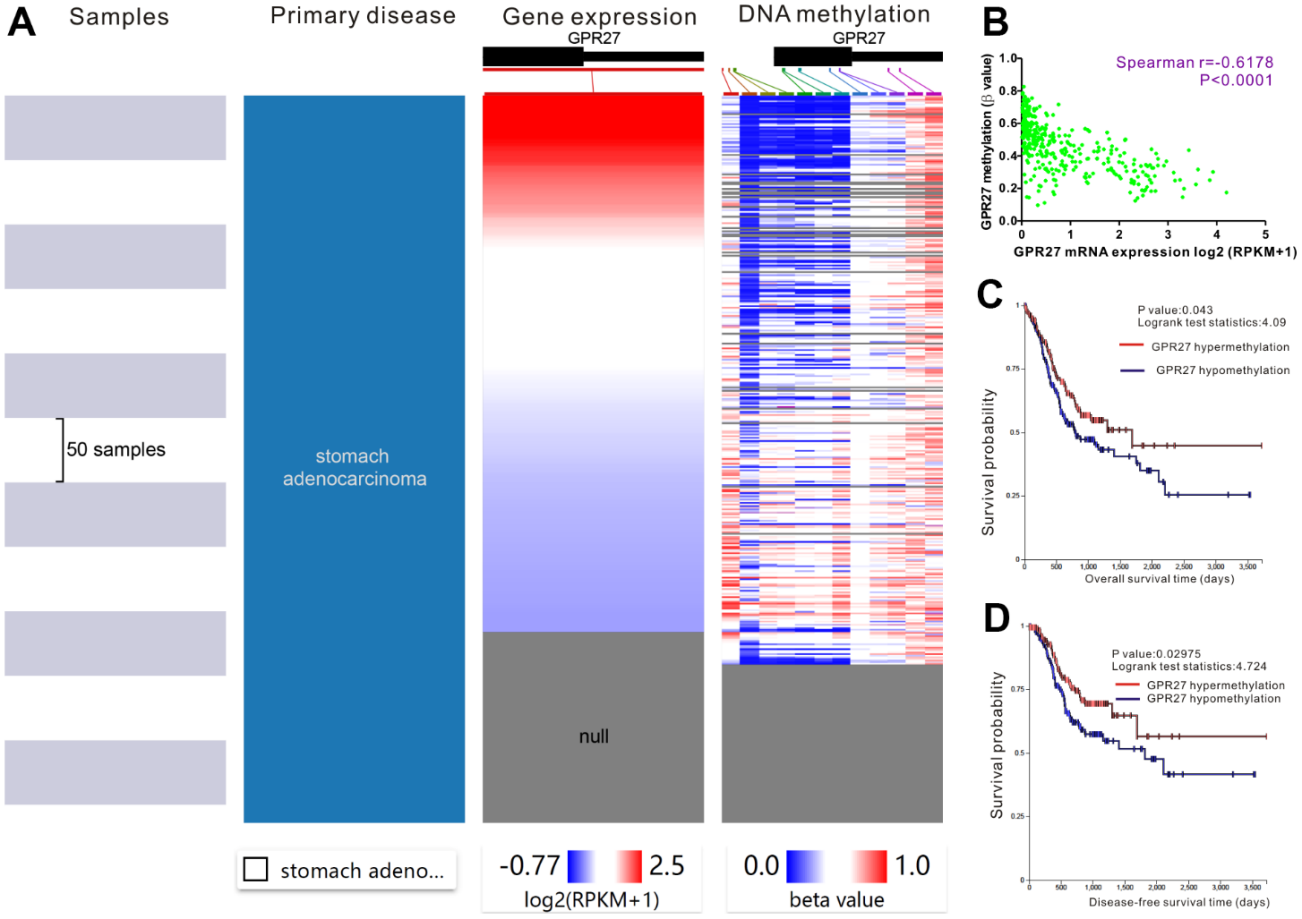
**Prognostic significance of GPR27 methylation in gastric cancer.** The heat map unveils that high expression of GPR27 corresponds to low DNA methylation in gastric cancer (**A**). A strong correlation between GPR27 expression and DNA methylation is noticed in gastric cancer (**B**). Hypermethylation of GPR27 is correlated with relatively superior overall survival (**C**) and disease-free survival (**D**) in sufferers with gastric cancer.

We applied the UCXC Xena to investigate the epigenetic regulation of GPR27 in GC patients and found that the methylation level of the GPR27 promoter was inversely linked to the transcription volume of GPR27 (Figure 3A). Therefore, Spearman correlation analysis was employed to examine the linkage between GPR27 promoter methylation level and GPR27 transcription level. There was a negative correlation (r=-0.6178 P<0.0001) between GPR27 promoter methylation and GPR27 transcription in GC (Figure 3B). Moreover, survival analysis was performed and results show that hypermethylation of GPR27 is correlated with relatively superior OS and DFS in GC patients (Figure 3C). Spearman correlation analysis was employed to evaluate the linkage between GPR27 mRNA level and 12CpG sites of GPR27 DNA promoter in GC. As shown in the correlation plots (Supplementary Figure 3), the cg22823146CpG site exhibited the strongest association with GPR27 mRNA level in GC (r=-0.6639, P<0.0001). We also explored the associations of the 6 CpG sites with clinical characteristics, including ethnicity, sex, age and prognosis (Supplementary Figure 4A). We conducted survival analysis and results show that GC patients with hypermethylation of cg10172415, cg03024619 and cg13562542 exhibit better survival outcome than those with hypermethylation of GPR27 (Supplementary Figure 4B–4G).

### Correlation between GPR27 mRNA level and tumor-immune microenvironment

**Figure 4:**
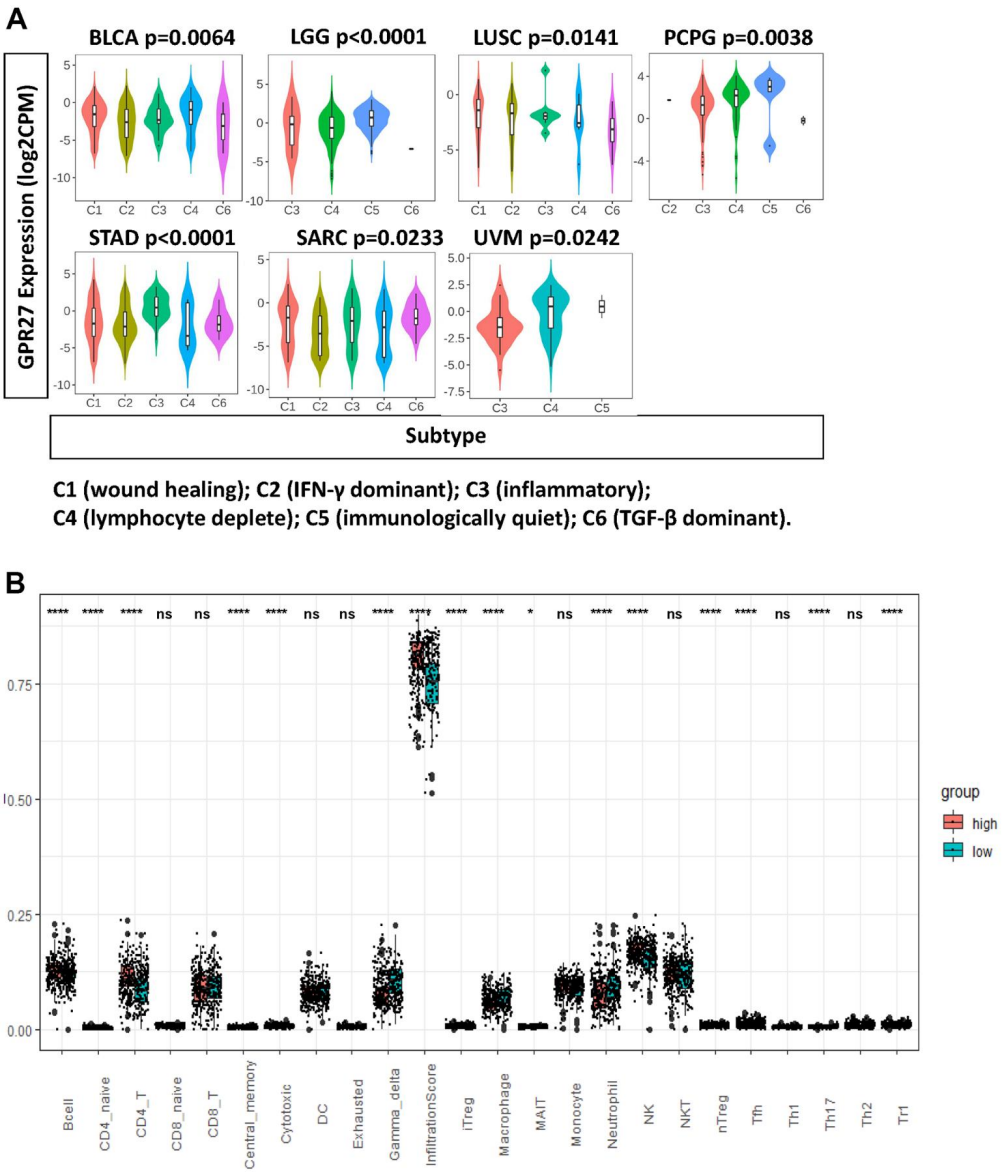
**GPR27 is associated with immune infiltration in several human cancers.** (**A**) GPR27 mRNA expression in different immune subtypes in BLCA, LGG, LUSC, PCPG, STAD, SARC, UVM from TISIDB (http://cis.hku.hk/TISIDB/index.php), (**B**) The fraction of tumor infiltrating immune cells in GPR27 high and low subgroups.

Using TISIDB, we analyzed the expression pattern of GPR27 mRNA levels in several immune subtypes. We found a significant difference in GPR27 expression pattern across immune subtypes in LGG, LUSC, BLCA, PCPG, STAD, SARC and UVM (Figure 4A). While in other cancers, we did not observe clear differences (Supplementary Figure 5).

To examine the association between GPR27 and immune cells, we applied ImmuCellAI to investigate the levels of 24 types of tumor infiltrating immune cells in GC. We found the proportion of B cells, CD4 naïve cells, CD4 T cells, central memory cells, iTreg cells, MAIT cells, nature killer cells, nTreg cells, Tfh cells, and TH17, Th2, and Tr1 cells was increased in the GPR27-high subgroup, whereas the proportion of neutrophils, macrophages, gamma delta cells, and cytotoxic cells was increased in the GPR27-low subgroup (Figure 4B).

**Figure 5:**
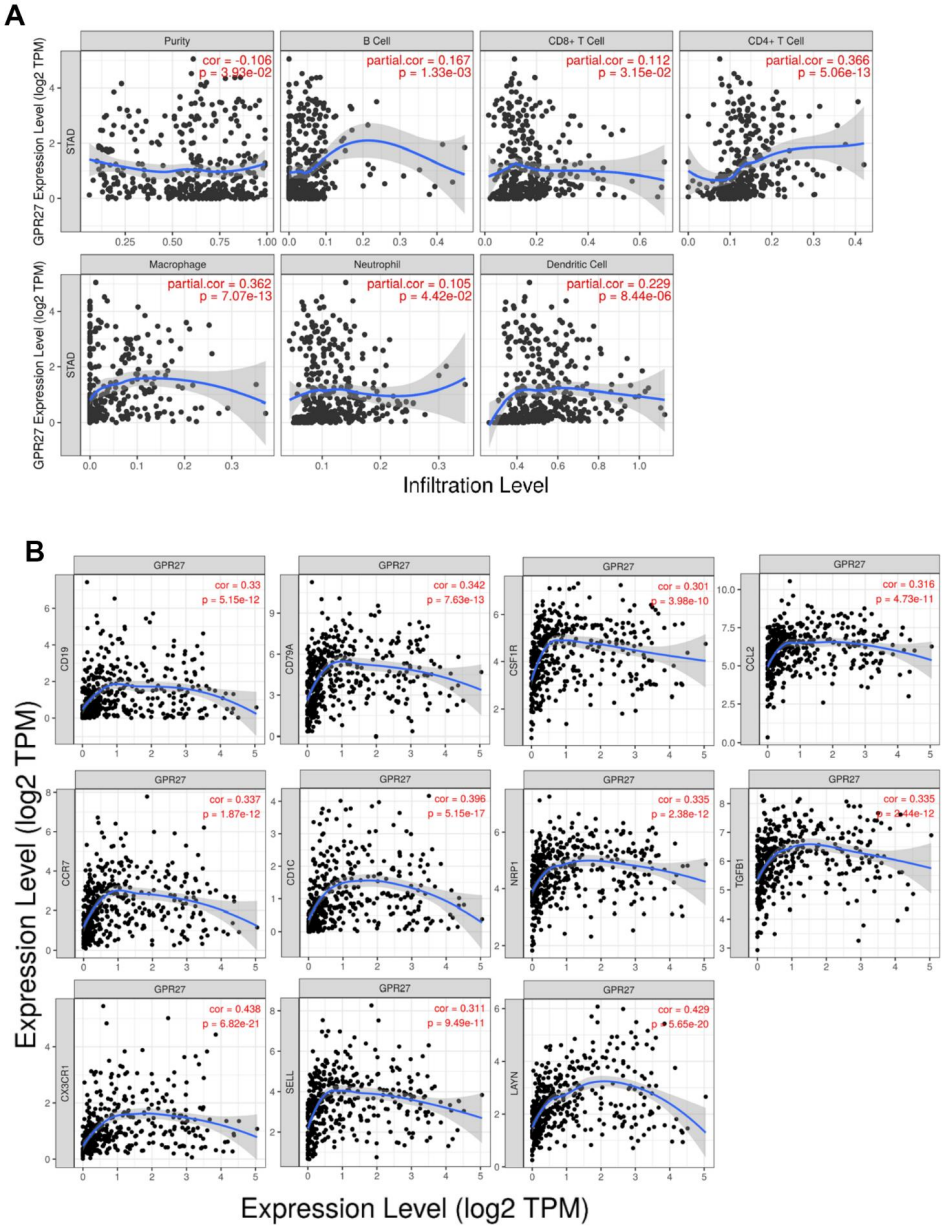
**Correlation between GPR27 and immune cells from TIMER (https://cistrome.shinyapps.io/timer/).** (**A**) The correlation of the expression of GPR27 with the infiltration of different immune cells. (**B**) The correlation of the expression of GPR27 and gene markers of immune cells.

We examine the association between GPR27 mRNA levels and six types of immune cells, and found that GPR27 mRNA level is positively linked to the infiltration of all those six types of immune cells in GC (Figure 5A). We examined the linkage between GPR27 mRNA level and various immune characteristics in GC. The genes listed in Tables 2, 3 were the markers of corresponding immune cells. Results show that GPR27 mRNA level was remarkably correlated with majority markers of immune cells in GC (Figure 5B). Finally, for the purpose of which cells express GPR27, we mined the human protein atlas database. Single-cell sequencing analysis reveals that GPR27 is mainly expressed in macrophages, followed by gastric mucus secreting cells (Supplementary Figure 6).

**Table 2:** Correlation analysis between GPR27 and gene markers of immune cells in TCGA-STAD.

**Description**	**Gene markers**	**None**			**Purity**	
		**Cor**	**p**		**Cor**	**p**
**B cell**	CD19	0.33	***		0.333	***
	CD79A	0.342	***		0.332	***
**T cell (general)**	CD3D	0.122	*		0.109	*
	CD3E	0.171	***		0.161	**
	CD2	0.153	**		0.142	**
**CD8+ T cell**	CD8A	0.152	**		0.138	**
	CD8B	0.176	***		0.183	***
**Monocyte**	CD86	0.148	**		0.132	**
	CSF1R	0.301	***		0.291	***
**TAM**	CCL2	0.316	***		0.317	***
	CD68	0.138	**		0.136	**
	IL10	0.195	***		0.189	***
**M1**	IRF5	0.254	***		0.268	***
	PTGS2	0.147	**		0.153	**
**M2**	NOS2	-0.146	**		-0.155	**
	CD163	0.198	***		0.192	***
	VSIG4	0.217	***		0.219	***
	MS4A4A	0.236	***		0.232	***
**Neutrophils**	CEACAM8	-0.03	0.536		-0.039	0.446
	ITGAM	0.298	***		0.296	***
	CCR7	0.337	***		0.331	***
**Natural killer cell**	KIR2DL1	0	0.994		-0.0013	0.794
	KIR2DL3	-0.049	0.315		-0.086	0.0947
	KIR2DL4	-0.122	*		-0.135	**
	KIR3DL1	-0.045	0.361		0.029	0.572
	KIR3DL2	0.051	0.303		0.032	0.533
	KIR3DL3	-0.04	0.414		-0.054	0.295
**Dendritic cell**	KIR2DS4	0.001	0.986		-0.016	0.757
	HLA-DPB1	0.213	***		0.203	***
	HLA-DQB1	0.063	0.201		0.052	0.308
	HLA-DRA	0.1	*		0.082	0.109
	HLA-DPA1	0.176	***		0.165	**
	CD1C	0.396	***		0.396	***
	NRP1	0.335	***		0.323	***
	ITGAX	0.196	***		0.188	***

**Table 3:** Correlation analysis between GPR27 and gene markers of different types of T cells in TCGA-STAD.

**Description**	**Gene markers**	**None**		**Purity**	
		**Cor**	**p**	**Cor**	**p**
Th1	TBX21	0.162	***	0.146	**
	STAT4	0.222	***	0.212	***
	STAT1	-0.048	0.33	-0.054	0.298
	TNF	0.134	**	0.118	*
	IFNG	-0.134	**	-0.145	**
Th1-like	HAVCR2	0.127	**	0.118	*
	IFNG	-0.134	**	-0.145	**
	CXCR3	0.119	*	0.01	*
	BHLHE40	0.0066	0.177	0.084	0.103
	CD4	0.245	***	0.239	***
Th2	STAT6	0.215	***	0.221	***
	STAT5A	0.204	***	0.201	***
Treg	FOXP3	0.169	***	0.166	**
	CCR8	0.205	***	0.199	***
	TGFB1	0.335	***	0.321	***
Resting Treg	FOXP3	0.169	***	0.166	**
	IL2RA	0.127	**	0.111	*
Effector Treg T-cell	FOXP3	0.169	***	0.166	**
	CCR8	0.205	***	0.199	***
	TNFRSF9	0.16	**	0.162	**
Effector T-cell	CX3CR1	0.438	***	0.43	***
	FGFBP2	0.253	***	0.238	***
	FCGR3A	0.055	0.261	0.054	0.292
Naïve T-cell	CCR7	0.337	***	0.331	***
	SELL	0.311	***	0.316	***
Effector memory T-cell	DUSP4	-0.092	0.0601	-0.113	0.0272
	GZMK	0.246	***	0.233	***
	GZMA	-0.007	0.888	-0.023	0.653
Resident memory T-cell	CD69	0.243	***	0.242	**
	CXCR6	0.128	**	0.119	*
	MYADM	0.388	***	0.368	***
General	CCR7	0.337	***	0.331	***
memory T-cell	SELL	0.311	***	0.316	***
Exhausted T-cell	IL7R	0.28	***	0.267	***
	HAVCR2	0.127	**	0.118	*
	LAG3	0.032	0.517	0.024	0.644
	CXCL13	0.145	**	0.148	**
	LAYN	0.429	***	0.423	***

### Clinical validation with 97 GC cases

**Figure 6:**
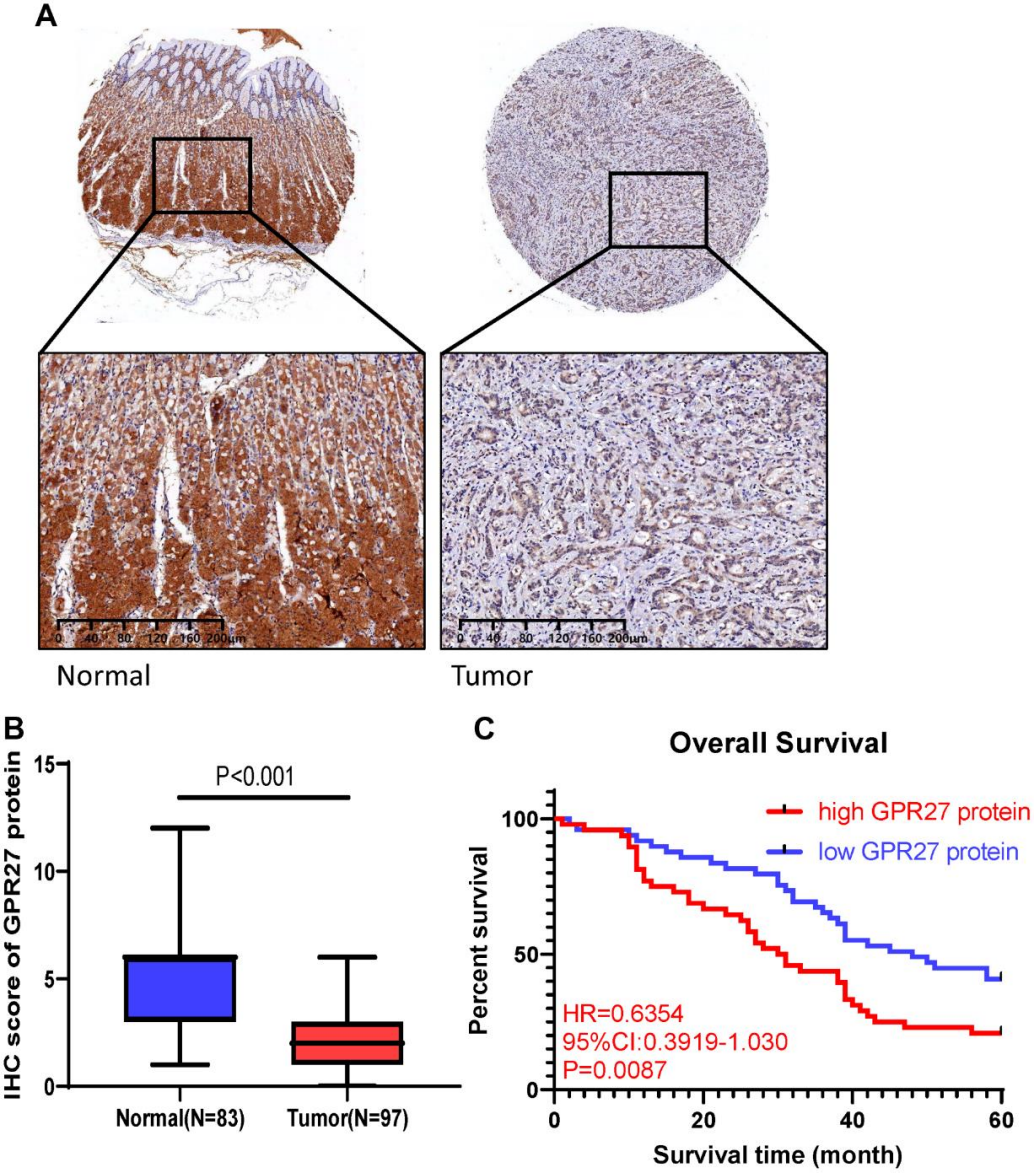
**GPR27 is downregulated in gastric cancer clinical samples and correlated with longer survival in gastric cancer patients.** (**A**) Immunohistochemical staining of normal and gastric cancer tissues with anti-GPR27 antibody. (**B**) Quantitative analysis of GPR27 staining shows significantly H-score in gastric tumor samples compared with adjacent normal tissues (83 normal tissues and 97 tumor samples). (**C**) Gastric cancer patients with GPR27 over-expression displayed less favorable overall survival than those with low GPR27 expression.

We utilized 97 GC patients to explore GPR27 protein level in GC patients. We performed semi-quantitative analysis and results showed a significant reduction in GPR27 staining intensity in GC tissues (Figure 6A, 6B). Furthermore, we utilized the Chi-square test to examine the clinical features between the GPR27 protein low and high group. As shown in Supplementary Table 2, significant differences were observed in tumor stage (P=0.0016) and distant metastasis (P=0.0335) between low-GPR27 and high-GPR27 groups.

Survival analysis showed that GC patients with lower GPR27 protein level had a longer OS times (HR=0.6354, 95%CI:03919-1.030, P=0.0087, Figure 6C). In conclusion, we conclude that high levels of GPR27 protein are a risk factor for poor prognosis in GC patients, which is consistent with our conclusions from the TCGA and GEO databases.

## DISCUSSION

Our analysis reveals the role of GPR27 in GC for the first time. Pan-cancer analysis revealed that GPR27 was abnormally expressed in most of malignant neoplasms, including GC. As for gene mutation, approximately 4% of GC patients exhibited genetic alterations in GPR27. We also found that most GPR27 mutations are missense mutations in GC, and this could partly explain its low expression in GC. Methylation analysis identified the strong reverse relationship between GPR27 expression and DNA methylation in GC (r=-0.6178, P<0.0001). Then, Kaplan-Meier survival analyses implicated that expression of GPR27, GPR27 methylation and GPR27 mutation were all correlated with the survival outcomes in sufferers with GC. More importantly, validation with clinical samples indicates that GPR27 protein was lowly expressed in GC specimens, which is consistent with the trend of GPR27 transcription. Survival curves also demonstrated that low expression of GPR27 protein is linked to superior overall survival rates and less recurrence among GC individuals. Collectively, our study substantiated that GPR27 is a reliable prognostic index in individuals with GC.

There are a large number of immune cells in the tumor microenvironment, which occupy a significant role in malignant tumors as a double-edged sword that inhibits or contributes to tumor progression [19]. Lymphocytes infiltrate tumor cells to regulate the immune response in GC. Some studies reached controversial results. For example, some experts deem that Tregs are protective, while others held the view that Tregs could inhibit the effector T cells which promotes the progression of GC [20]. High abundance of T cells in GC tissues are correlated with relatively favorable survival outcomes [21]. T regulatory cell is a member of tumor infiltrating lymphocytes, which could suppress the immune response mediated by CD8+ and CD4+ T cells, thus linked to undesirable survival outcomes [22]. As for B cell, B cells plays a critical role in the anti-tumor immune response via the secretion of antibodies and cytokines in GC. While, not all B cells positively regulate anti-tumor immune response in GC, regulatory B cells negatively regulate anti-tumor responses via the secretion of anti-inflammatory cytokines [23]. Mounting evidence has highlighted their clinical significance in the prediction of survival outcomes and immunotherapy efficacy [24]. Our analysis found that GPR27 expression was positively linked to majority of immune cells in GC tissues. Further enrichment analysis confirmed that GPR27 was primarily evolved in the activation of T cells., indicating that GPR27 plays a significant role in the adjustment of immune response in GC. In addition, GPR27 mRNA level was remarkably correlated with majority markers of immune cells in GC. Our results pointed out that GPR27 occupies a certain role in the adjustment of tumor immunity, and might be a novel target for immunotherapy in GC. Whereas, the accurate mechanisms of GPR27 in the TME still require thorough investigation.

DNA methylation is a covalent chemical modification, which plays a critical the role of DNA methylation in carcinogenesis and metastasis in malignant tumors [25, 26]. Recently, DNA methylation has been shown to have certain predictive significance in the survival assessment of GC patients. Dai et al. [27] designed a DNA methylation signature with seven significant genes, which is associated with survival outcomes of GC. Moreover, Li et al. and its coworkers [28] found that methylation of TGFβ2 could be used for the prognostic assessment among individuals with GC. A recent report clarified the DNA methylation driven gene signature (TUBB6, MICU3, PODN, MYO1A, NPY and RHOJ) is significantly linked to the long-term survival outcomes of GC patients [29]. Our analysis identified the strong negative relationship between GPR27 mRNA level and DNA methylation, and we deemed that the expression of GPR27 in GC is negatively linked to its DNA methylation. Fortunately, hypermethylation of GPR27 not only predicts relatively favorable overall survival but also predicts enhanced disease-free survival in GC.

We observed that GPR27 mRNA exhibits low expression level in GC tissues, which was in line with other GPCR member genes in cancer. GPR155 mRNA was suppressed in GC cell lines, and expression level of GPR155 in GC individuals were associated with distant metastasis and tumor recurrence [30]. Moreover, GPR68 is lowly expressed in GC [31]. However, survival analysis demonstrated that low expression of GPR27 mRNA is linked to better OS and DFS. Correlation analysis identified the reverse linkage between GPR27 mRNA level and TMB, which might be the part reason for its better prognosis in GC.

Recently, a high TBM has been demonstrated to strengthen clinical response to immunotherapy, including NSCLC patients [32] and melanoma [33]. Samstein et al. [34] have investigated TMB association with survival outcomes in individuals receiving immune checkpoint blockers (ICBs) therapy, and concluded that individuals with higher TMB exhibited better survival outcomes across various cancer types. Wang et al. [35] deemed that high TMB may be a reliable predictive biomarker for favorable OS of GC patients receiving ICB. Exploration of the relationship between TMB and the mutations of key genes is needed to guide immunotherapy for GC. Given our analysis, we speculated that GC patients with low expression of GPR27 possessed higher TMB, and a high TBM is directly correlated to a good treatment response to immunotherapy among GC patients [36]. Taken these together, we concluded that GPR27 may influence the prognosis outcomes of GC patients partly as a result of TBM.

Our research cast light on the role of GPR27 in GC, but our analysis still has two limitations. Firstly, we systematically explored the mRNA expression, DNA methylation, gene mutation and TMB of GPR27 in GC, and validated the bioinformatics conclusion with clinical cohort data, but this study does not explain the mechanism of GPR27 in gastric cancer cell’s growth and metastasis. Additionally, the amount of GC patients in the clinical validation was limited to 97 cases, and information regarding immunotherapy was unavailable. We could not assess the predictive value of GPR27 for immunotherapy efficiency. Hence, future analysis to validate the biological functions of GPR27 and predictive values for immunotherapy response in GC is urgently needed.

## CONCLUSIONS

We comprehensively investigated GPR27 mRNA expression, DNA methylation, TBM, prognostic significance, protein expression, and correlation with tumor-infiltrating immune cells based on multi-omic bioinformatics and clinical cohort data. GPR27 is a reliable clinical prognostic index for GC sufferers, and may become a new target for GC immunotherapy. Further biological researches related to GPR27 in GC is warranted to validate our present findings.

## Supplementary Material

Supplementary Figures

Supplementary Tables
