# Supplementary material for: Prognostic significance and immune characteristics of GPR27 in gastric cancer
Source: Aging (Albany NY). 2023 Sep 12;15(17):9144–66. doi: 10.18632/aging.205023 (PMC10522374; doi:10.18632/aging.205023)
Supplement: Supplementary Figures [file aging-15-205023-s001.pdf]

SUPPLEMENTARY FIGURES

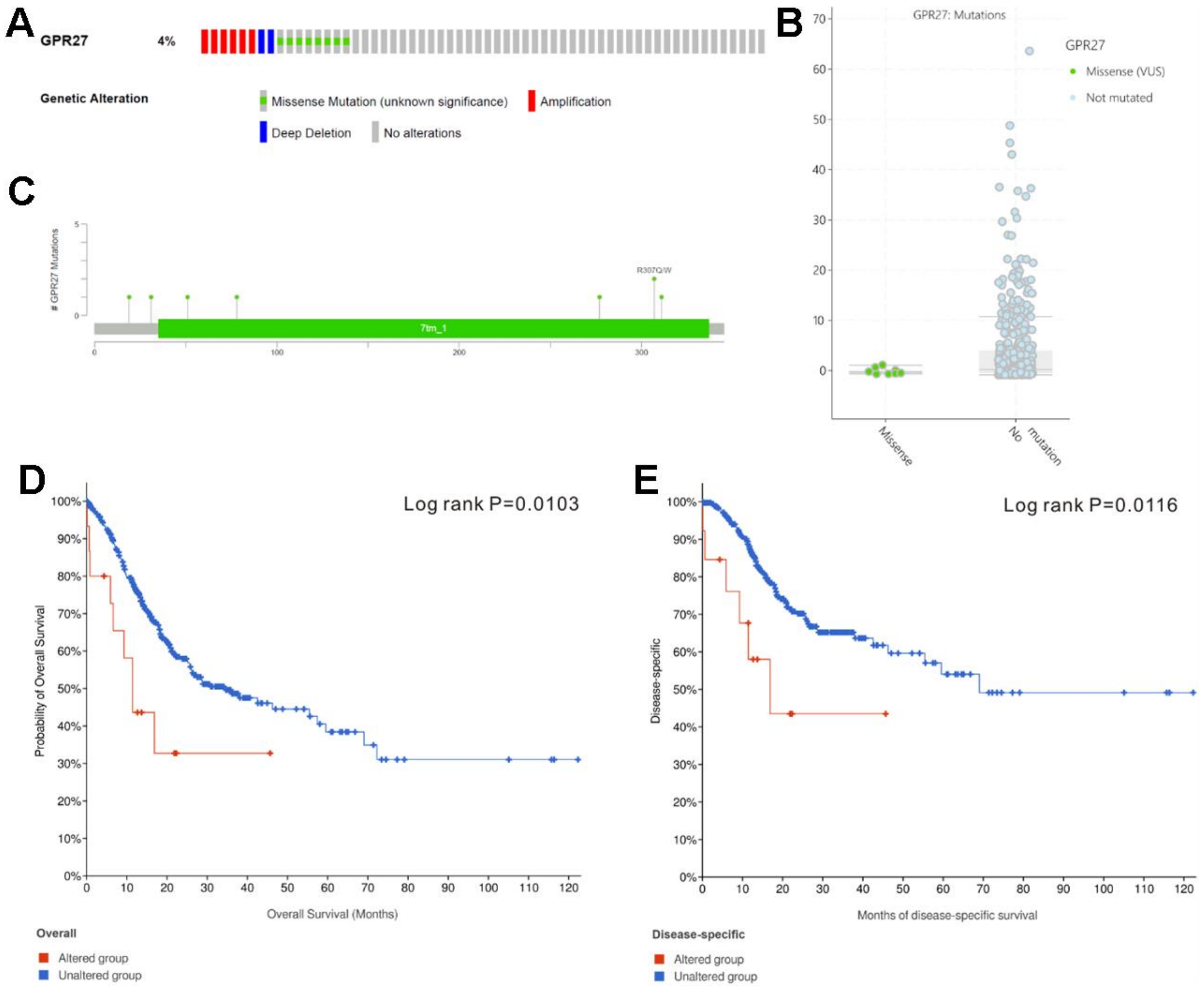

Supplementary Figure 1. Mutation types of GPR27 and survival analysis of GPR27 mutation in gastric cancer. (**A**) The mutation frequency of GPR27 in gastric cancer. (**B**) GPR27 mutation diagram circles are colored with respect to the corresponding mutation types. (**C**) Correlation between GPR27 expression and GPR27 mutation. GPR27 mutation predicts worse overall survival (**D**) and less favorable disease-free survival (**E**).

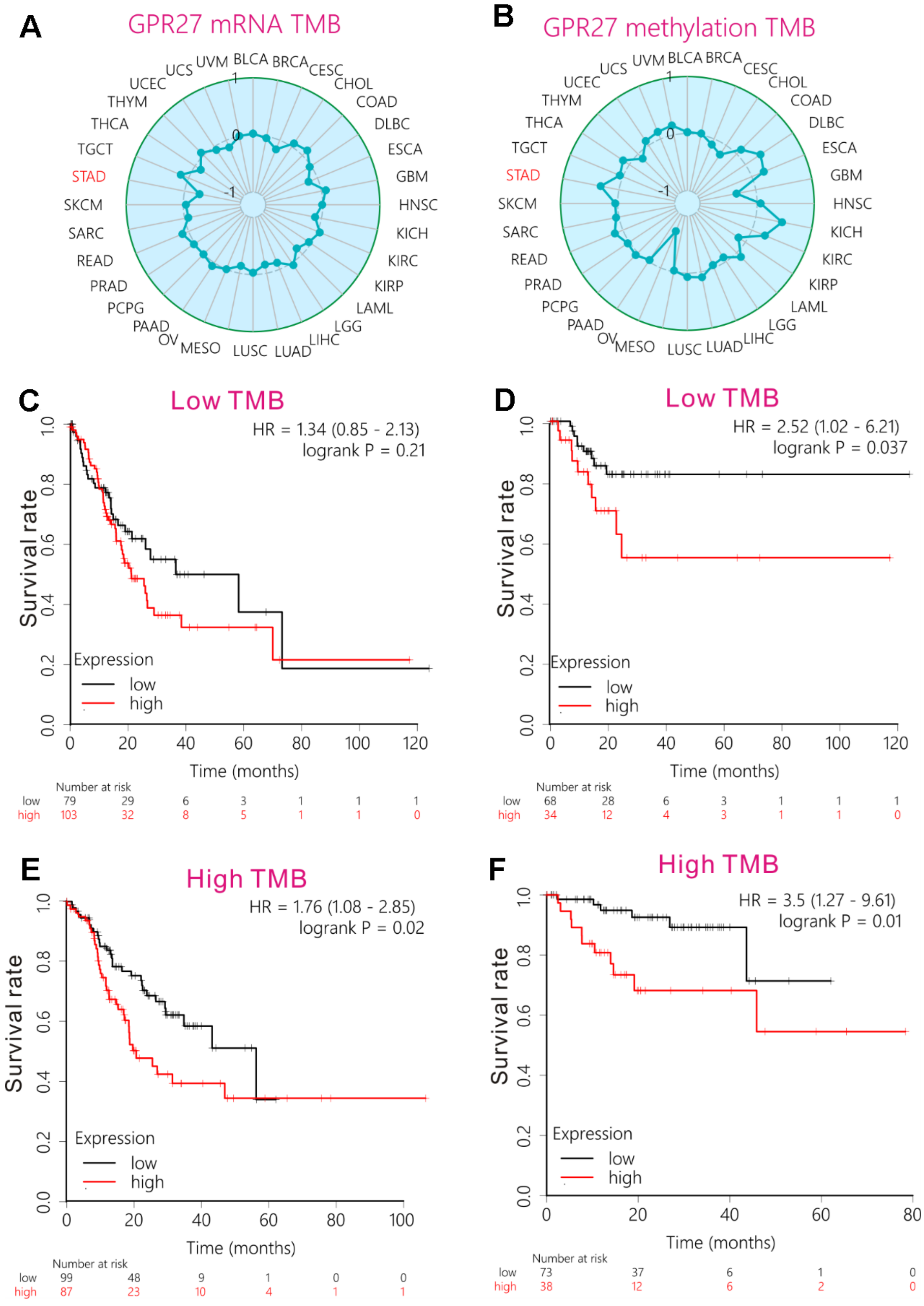

Supplementary Figure 2. Subgroup survival analysis stratified by tumor mutation burden in gastric cancer. Expression of GPR27 is inversely correlated with tumor mutation burden (**A**), and DNA methylation of GPR27 is positively correlated with tumor mutation burden in gastric cancer (**B**). Correlation between GPR27 expression and overall survival was not observed in gastric cancer individuals with low tumor mutation burden (**C**), but in individuals with high tumor mutation burden (**D**). Correlation between GPR27 expression and overall survival not only existed in gastric cancer individuals with low tumor mutation burden (**E**), but also in individuals with high tumor mutation burden (**F**).

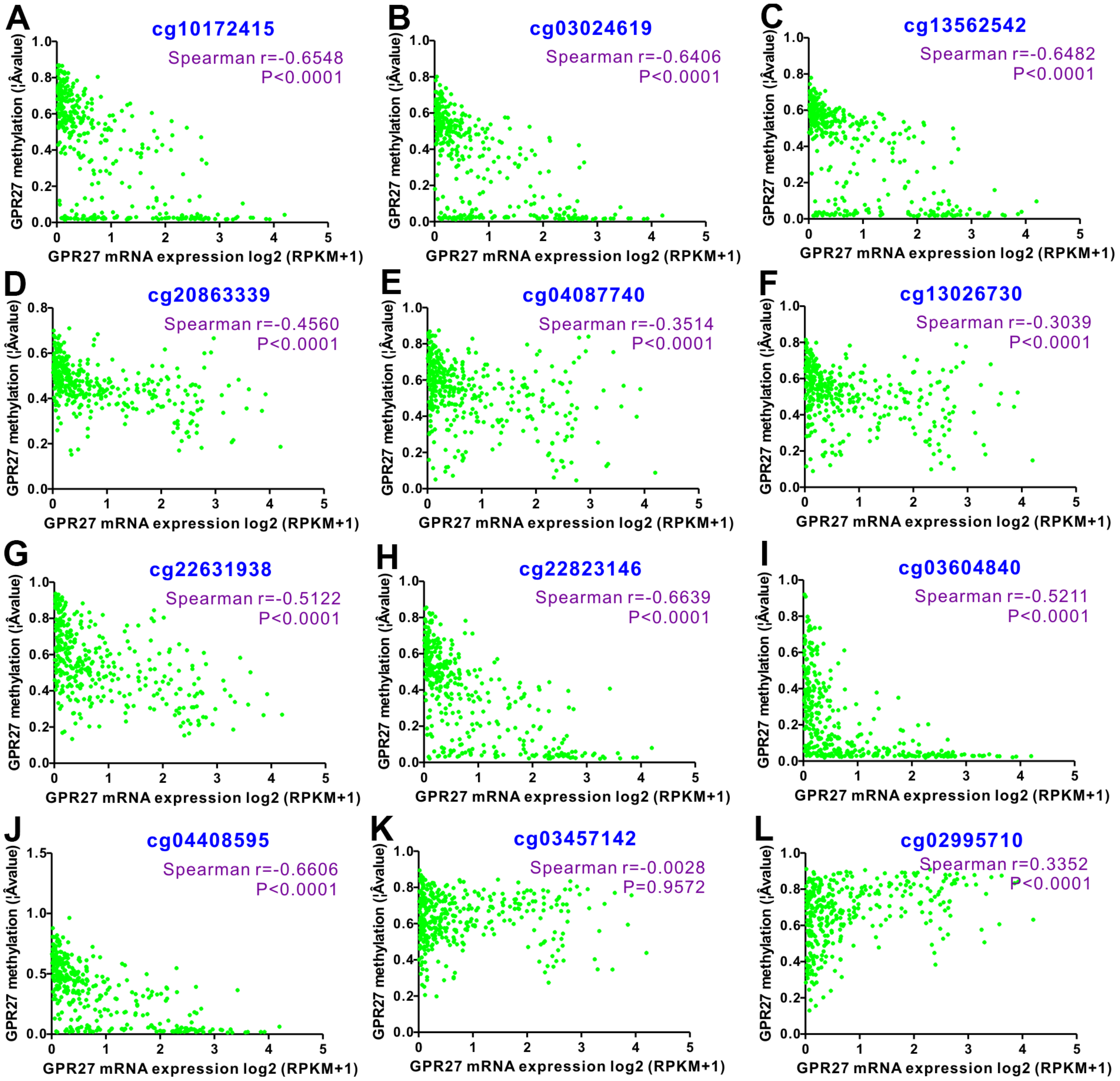

Supplementary Figure 3. Relationship between GPR27 expression and 12 CpG sites of GPR27 DNA promoter in gastric cancer. (**A**) cg10172415, (**B**) cg03024619, (**C**) cg13562542, (**D**) cg20863339, (**E**) cg04087740, (**F**) cg13026730, (**G**) cg22631928, (**H**) cg22823146, (**I**) cg03604840, (**J**) cg04408595, (**K**) cg03457142, (**L**) cg02995710.

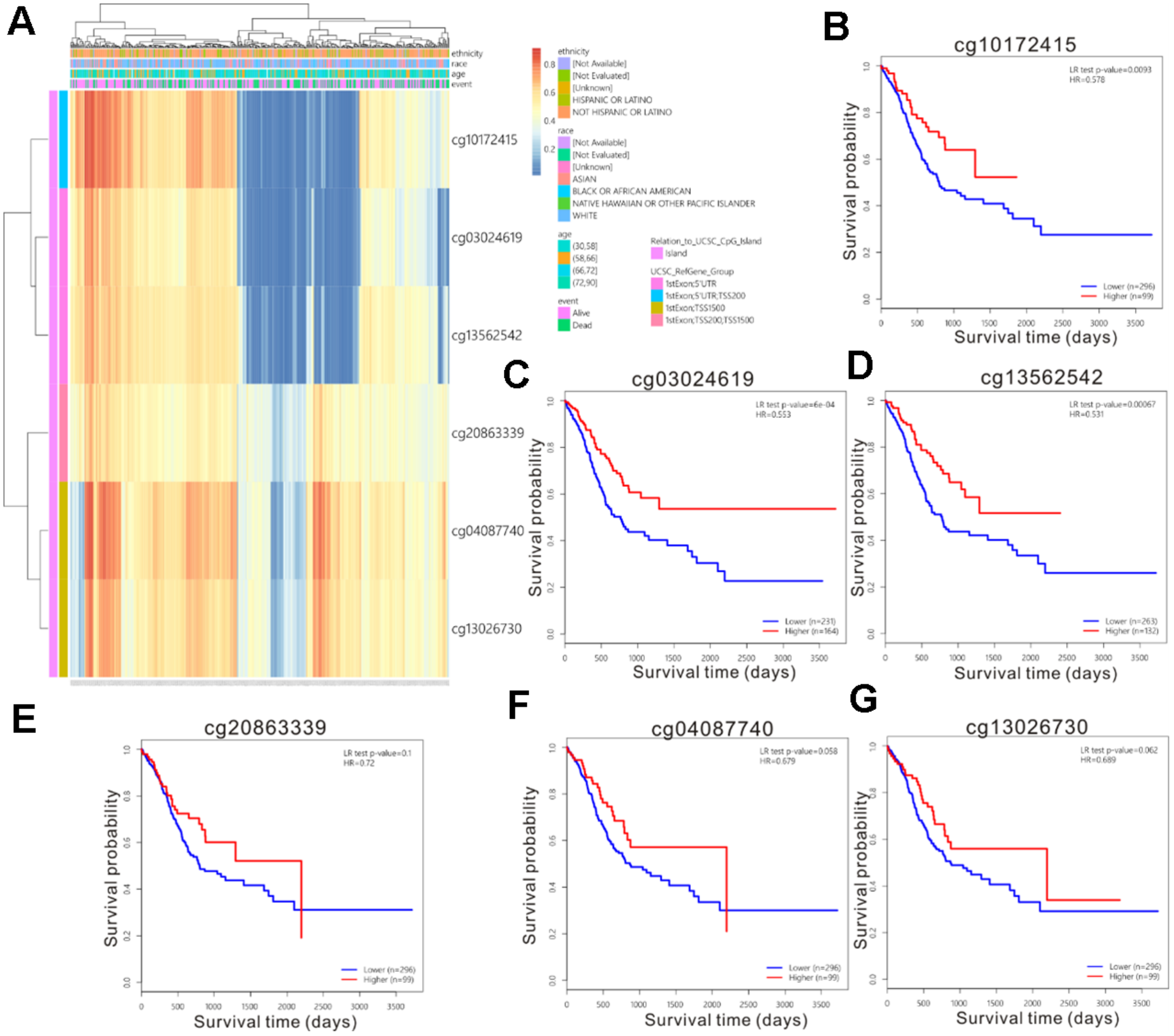

Supplementary Figure 4. Clinical significance and prognostic value of DNA methylation in gastric cancer. (**A**) heat map of correlation between GPR27 methylation and clinical metrics. Survival analysis of six CpG sites of GPR27 DNA methylation in gastric cancer ((**B**) cg10172415, (**C**) cg03024619, (**D**) cg13562542, (**E**) cg20863339, (**F**) cg04087740, (**G**) cg13026730).

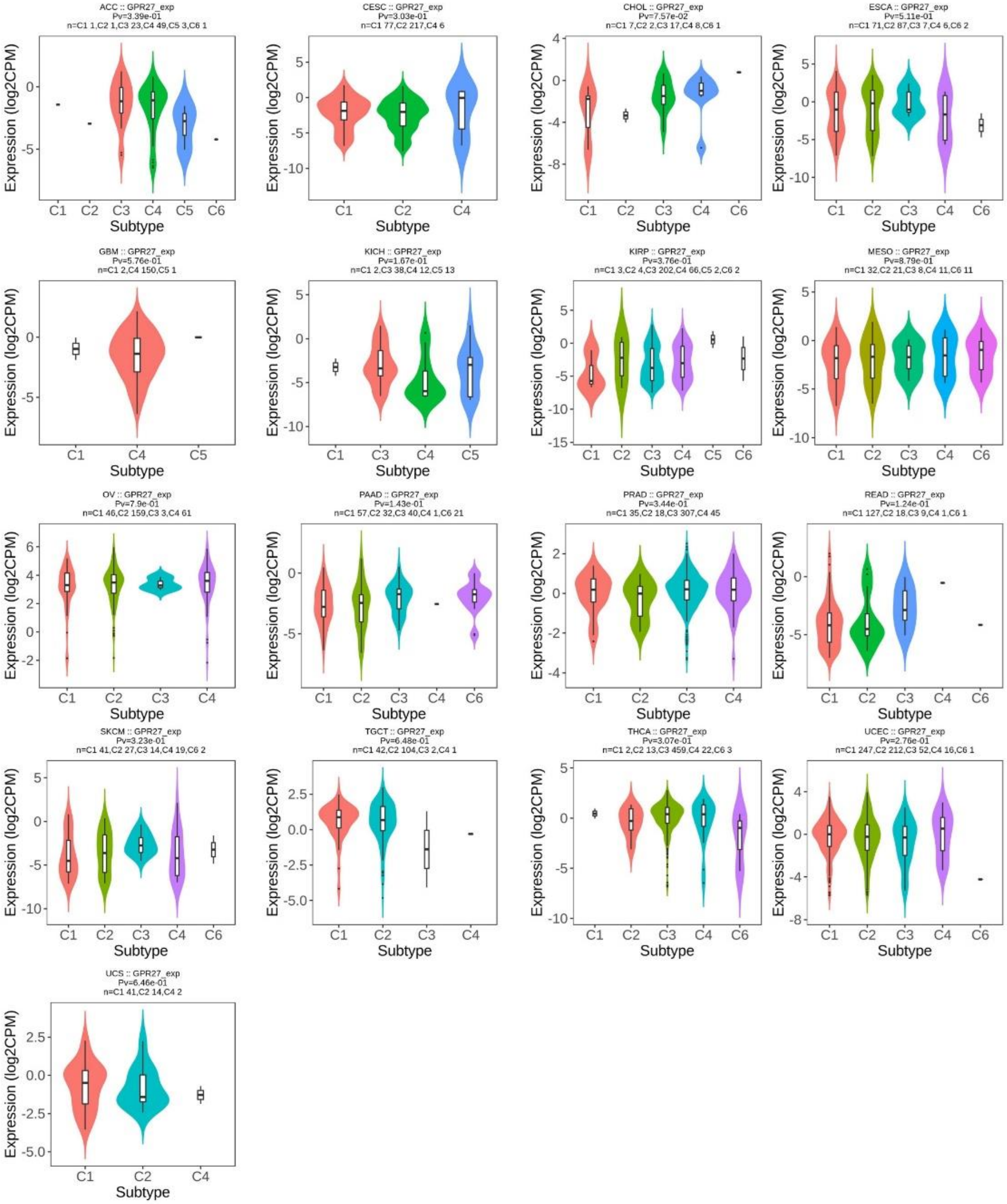

Supplementary Figure 5. Difference of GPR27 expression in different immune subtypes. The expression of GPR27 had no significantly statistical difference across different immune subtypes in these cancers.

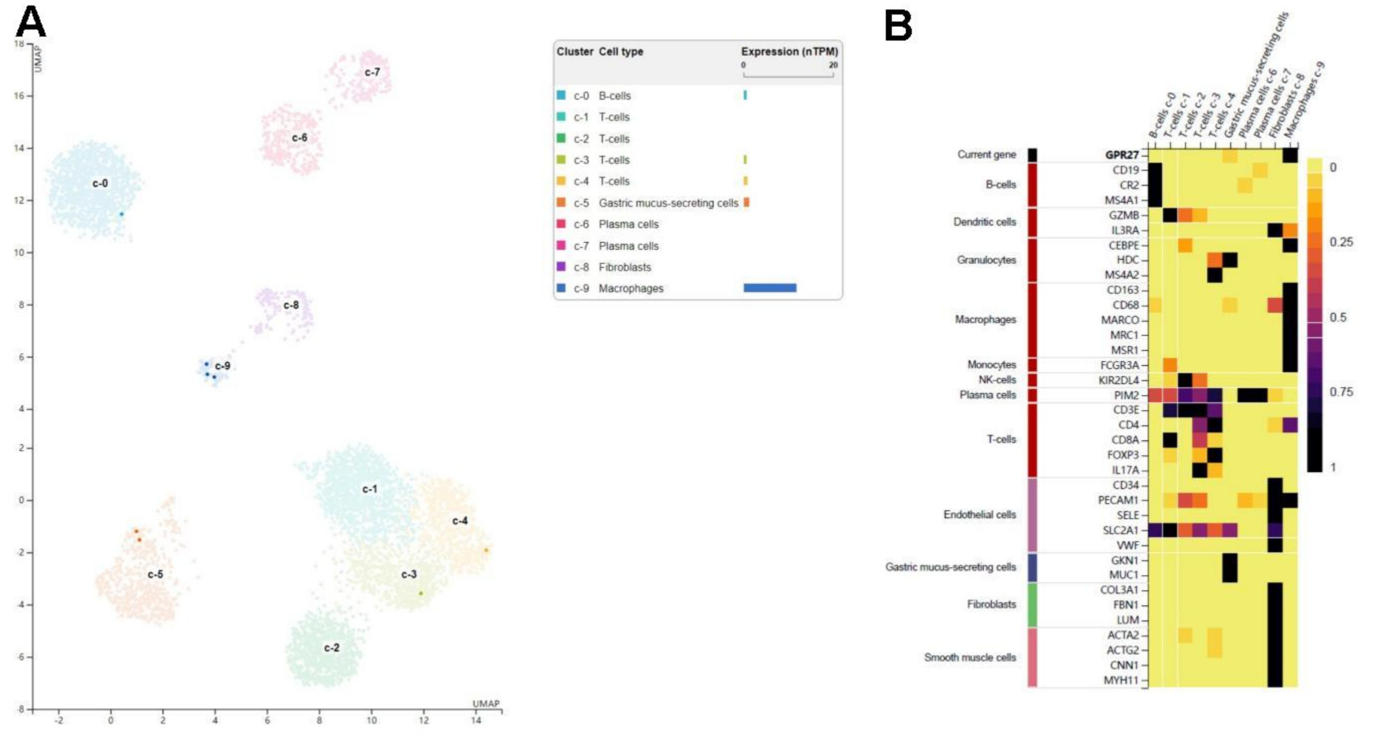

Supplementary Figure 6. GPR27 expression in gastric cells and immune cells. (**A**) Cluster cell type analysis in GC. (**B**) Expression of GPR27 mRNA in gastric cells and immune cells.
