# Supplementary material for: Prognostic significance and immune characteristics of GPR27 in gastric cancer
Source: Aging (Albany NY). 2023 Sep 12;15(17):9144–66. doi: 10.18632/aging.205023 (PMC10522374; doi:10.18632/aging.205023)
Supplement: Supplementary Tables [file aging-15-205023-s002.pdf]

SUPPLEMENTARY TABLES

Supplementary Table 1. Correlation between GPR27 mutation and clinical features.

Clinical attribute	Attribute type	Statistical test	P value
Tissue Source Site Code	Sample	Chi-squared Test	2.53E-03
Tissue Source Site	Sample	Chi-squared Test	2.53E-03
TMB	Sample	Wilcoxon Test	7.52E-03
Mutation Count	Sample	Wilcoxon Test	0.0184
MSIsensor Score	Sample	Wilcoxon Test	0.0225
In PanCan Pathway Analysis	Patient	Chi-squared Test	0.0382
Sex	Patient	Chi-squared Test	0.0466
Subtype	Patient	Chi-squared Test	0.0469

Supplementary Table 2. Statistical analysis of the protein of GPR27 and different clinical features of gastric cancer.

Clinical features	GPR27 protein level		χ^2	P
	Low	High		
Gender				
male	35	39	1.293	0.2555
female	14	9		
Age				
≤55	18	15	0.325	0.5686
>55	31	33		
Tumor size (cm3)				
≤35	43	39	0.7849	0.3756
>35	6	9		
Tumor stage				
T1+T2	20	6	9.909	0.0016
T3+T4	29	42		
Distant metastasis				
M0	46	38	4.521	0.0335
M1	3	10		
Lymph node metastasis				
N0+N1	22	19	0.2807	0.5963
N2+N3	27	29		
